# Upregulated Expression of CYBRD1 Predicts Poor Prognosis of Patients with Ovarian Cancer

**DOI:** 10.1155/2021/7548406

**Published:** 2021-09-21

**Authors:** Rui Chen, Jianhong Cao, Wei Jiang, Shunli Wang, Jingxin Cheng

**Affiliations:** ^1^Department of Gynecology, East Hospital Affiliated to Tongji University, Shanghai 200012, China; ^2^Department of Heart Failure, East Hospital Affiliated to Tongji University, Shanghai 200120, China; ^3^Department of Pathology, East Hospital Affiliated to Tongji University, Shanghai 200120, China

## Abstract

Cytochrome b reductase 1 (CYBRD1) promotes the development of ovarian serous cystadenocarcinoma (OV). We assessed the function of CYBRD1 in OV underlying The Cancer Genome Atlas (TCGA) database. The correlation between clinicopathological characteristics and CYBRD1 expression was estimated. The Cox proportional hazards regression model and the Kaplan–Meier method were applied to identify clinical features related to overall survival and disease-specific survival. Gene set enrichment analysis (GSEA) was applied to identify the relationship between CYBRD1 expression and immune infiltration. CYBRD1 expression in OV was significantly associated with poor outcomes of primary therapy and FIGO stage. Patients with high levels of CYBRD1 expression were prone to the development of a poorly differentiated tumor and experience of an unfavorable outcome. CYBRD1 expression had significant association with shorter OS and acts as an independent predictor of poor outcome. Moreover, enhanced CYBRD1 expression was positively associated with Tem, NK cells, and mast cells but negatively associated with CD56 bright NK cells and Th2 cells. CYBRD1 expression may serve as a diagnostic and prognostic indicator of OV patients. The mechanisms of poor prognosis of CYBRD1-mediated OV may include increased iron uptake, regulation of immune microenvironment, ferroptosis related pathway, and ERK signaling pathway, among which ferroptosis and ERK signaling pathway may be important pathways of CYBRD1-mediated OV. Furthermore, we verified that CYBRD1 was upregulated in OV and significant correlated with lymph nodes metastasis, advanced stage, poor-differentiated tumor, and poor clinical prognosis in East Hospital cohort. The results of this study may provide guidance for the development of optimal treatment strategies for OV.

## 1. Introduction

Ovarian cancer, the sixth most common genital malignancy among women worldwide, is the most lethal gynecological tumor [1]. Ovarian cancer features extensive peritoneal spreading, and 70% of patients are first diagnosed at a late stage, most frequently with serous carcinoma [2]. Serous ovarian cancer (OV) accounts for over 70% of deaths of patients with ovarian cancer, and overall survival has not changed significantly for fifty years. According to the World Health Organization, 230,000 new cases of OV are diagnosed annually, and 50,000 women die each year [3]. Most patients with advanced OV experience a 29% 5-year survival rate compared with 92% at an early stage [2]. Despite the highly malignant phenotype and complex pathogenesis of OV, the molecular mechanism is not understood. Therefore, it is critically important to identify prognostic indicators of the progression of OV. The molecular characteristics of OV include genomic instability and clonal diversity [4, 5]. Even when treated with an inhibitor of ADP-ribose polymerase, OV remains incurable and lethal [6]. Extensive studies show that apoptotic Treg cell-mediated immunosuppression correlates with poor prognosis [7]. Further, specific widespread patterns of intraperitoneal dissemination of tumor cells contribute to the heteromorphosis of the immune microenvironment [8]. Numerous studies support the conclusion that tumor-infiltrating lymphocytes (TILs) [6] influence clinical outcomes of patients with OV [8]. Moreover, TILs may contribute to tumor progression [8], the therapeutic efficacy of PD-L1 [9], and the prognostic implications of neoadjuvant chemotherapy [10]. These findings underscore the significance of immune microenvironments associated with OV. Therefore, we suspected that the expression of CYBRD1 might regulate OV invasion and metastasis through the immune microenvironment.

Cytochrome b reductase 1 (CYBRD1) is an iron-regulated ferric reductase that mediates iron-regulated signaling pathways [11] by catalyzing the conversion of ferric to ferrous ion during iron absorption [12]. Ferrous iron promotes DNA damage and participates in the pathogenesis and progression of cancer by inducing the production of reactive oxygen species [13, 14]. The loss of ferrous ion binding leads to the apoptotic death (ferroptosis) of hepatic cancer cells that is mediated by DNA damage induced by procaspase-3-activating compound 1 (PAC-1) [14]. Ferroptosis (iron-regulated cell death) contributes to the maintenance of the stability of the tumor microenvironment [13]. Further, CYBRD1 is expressed at higher levels in tumors of patients with breast cancer than those of normal tissues, and high levels of CYBRD1 play a role in prolonging survival by inhibiting FAK activation [15]. These findings support the conclusion that CYBRD1 expression shows promise as a predictor of prognosis. However, insufficient data are available to link CYBR1D to the absorption of ferrous ions and its association with the immune microenvironment.

To address these unanswered questions, here, we aimed to assess the prognostic value of CYBRD1 expression in the immune tumor microenvironment of OV through analysis of gene expression profiles obtained from The Cancer Genome Atlas (TCGA) (https://tcga-data.nci.nih.gov/tcga/) [16]. To further investigate the mechanisms and understand the biological pathways underlying OV, we conducted gene set enrichment analysis (GSEA) to identify pathogenic genes whose products participate in a CYBRD1-associated regulatory network. We further analyzed TCGA data to determine the effect of CYBRD1 on the clinical outcomes of patients with OV and to identify relevant signal transduction pathways associated with CYBRD1 function that contribute to the malignant phenotype of OV. We made correlation analysis of the correlation between CYBRD1 expression and immunocytes and ferroptotic markers. Moreover, we prove a correlation between CYBRD1 and clinicopathological variables and draw survival curves to analyze the correlation between CYBRD1 expression with OS in patients with ovarian cancer from Shanghai East Hospital (EH). Our results suggest that CYBRD1 expression is closely correlated with the prognosis of patients. The results provide insights into the mechanism of CYBRD1 function in OV.

## 2. Materials and Methods

### 2.1. Data Acquisition and Bioinformatics Analysis

RNA-seq data (376 patients with OV (workflow type: HTSeq counts)) and relevant clinical data were obtained from TCGA. RNA-seq data were obtained using an Illumina next-generation sequencing platform. Clinical data included histological grade, clinical stage, and anatomical locations. We acquired primary outcomes of therapy, overall survival (OS), and disease-specific survival (DSS) to analyze clinical prognosis. The inclusion criteria were (a) clinical stages I–IV, (b) complete follow-up data, and (c) microarray-based expression data. Gene expression values are expressed as log2. The correlation between CYBRD1 expression and clinicopathological variables was analyzed from 100 patients diagnosed with OV from EH cohort between 2010 and 2020. Samples with absent or unavailable clinical indicators were treated as missing values. Consent was obtained from the study participants prior to study commencement. All experiments were approved by the Ethics Committee of the Tongji University Animal Ethics Committee (Shanghai, China).

### 2.2. Gene Set Enrichment Analysis

We used GSEA [17] to investigate the expression of CYBRD1 in OV. CYBRD1 expression data were stratified into low and high types to annotate biological functions (1000 permutations), and reactome pathways (reactome.org) were illustrated using cluster Profiler [18] (*P* < 0.01).

### 2.3. Analysis of Immune Infiltration and Ferroptosis

We used marker genes of 24 types of immune cells described by Bindea et al. [19] to conduct gene set enrichment analysis (ssGSEA) to evaluate 24 types of tumor-infiltrating immune cells (TIICs) [17]. We used MaxStat (R package) [20] to stratify TIICs into low- and high-abundance groups. Furthermore, we analyzed the correlation between CYBRD1 expression with ferroptotic biomarkers (BECN1, FLT3, VDAC2, ALOX12, ACSL4, and GPX4). Gene expression data were normalized and analyzed using GSVA (R package) [21]. ssGSEA classifies gene sets associated with biological function, chromosomal localization, and physiological regulation [18]. The significance of the correlation between CYBRD1 and TIICs and ferroptotic biomarkers in OV was evaluated using Spearman's rank correlation analysis. An FDR <0.25 and adjusted *P* value <0.05 were set as the threshold values.

### 2.4. Immunohistochemistry

All samples were fixed in 4% paraformaldehyde at 4°C overnight. Five-micrometer-thick histological sections were processed by ethanol dehydration, xylene clearing, and paraffin embedding. Each section was stained with hematoxylin and eosin. Sections were incubated with primary antibodies (anti-CYBRD1; 1 : 500, Bioss, China) at 4°C overnight. The staining procedure was performed according to the instruction of the commercial kit (ZsBio, China).

IHC analysis was performed by two independent pathology investigators at 400× magnification in five randomly selected representative fields separately. A quantitative scoring system was applied to the assessment [22]. The staining intensity criteria were as follows: no positive coloring count 0 points, light yellow (weak positive) count 1 points, brown yellow (positive) count 2 points, and brown (strong positive) count 3 points. Expression intensity = staining intensity × percentage of positive cells [23]. ImageJ software was used to measure the grayscale value of the exposure slices to calculate the protein expression (semiquantitative), and the expression of CYBRD1 was divided into high-expression group and low-expression group.

### 2.5. Statistical Analysis

Survival rates analysis was performed to estimate the association of the OS and DSS of OV patients in the CYBRD1^low^ and CYBRD1^high^ groups using the Kaplan–Meier method and Cox regression. We then estimated the predictive performance of CYBRD1 on clinical prognosis (including OS and DSS), as well as other clinicopathological features using univariate and multivariate Cox regression analysis.

All experimental errors are shown as two standard error of the mean (representing 95% confidence intervals). Patients' survival rates were estimated using the Kaplan–Meier method. Survival curves were assessed using the log-rank test. We used the Mann–Whitney *U* test to evaluate the correlation between CYBRD1 expression and clinicopathological variables. A set of 376 OV samples were divided into CYBRD1^low^ and CYBRD1^high^ groups to determine the potential relevance of OS to clinical features. Clinicopathological variables of the CYBRD1^low^ and CYBRD1^high^ groups were subjected to logistic regression analysis. Multivariate analyses using the Cox proportional hazards model were conducted to estimate DSS and OS while adjusting for potential confounders. The hazard ratio (HR) and 95% confidence interval (CI) were calculated for each variable. Comparison between categorical variables was made using an *χ*2 analysis. Statistical analyses were conducted using the SPSS software (version 22.0), and *P* < 0.05 indicates a significant difference. The median value of the CYBRD1 expression was defined as the cutoff value. R language 3.6.1^2^ was used to conduct these analyses. The significance of the association between TIICs, ferroptotic biomarkers, and CYBRD1 expression in OV was evaluated using Spearman rank correlation analysis.

To provide reliable evidence of the predictive value of CYBRD1 for patients with OV in EH cohort, a nomogram and calibration that integrated the CYBRD1 and independent risk factors was constructed to predict the 1-year, 3-year, and 5-year OS for OV patients in East Hospital cohort.

## 3. Results

### 3.1. Patients' Clinicopathological Characteristics

We selected 376 primary samples with array-based TCGA gene expression data and determined the stage-specific distribution of patients' clinical variables (Table 1). All patients were divided into CYBRD1 low-expression group (L group) and high-expression group (H group). Disease stages were as follows: stage I, L group, *n* = 1 (0.3%); stage II, L group, *n* = 11 (2.9%) and H group, *n* = 11 (2.9%); stage III, L group, *n* = 145 (38.9%) and H group, *n* = 148 (39.7%); and stage IV, L group, *n* = 30 (8.0%) and H group, *n* = 27 (7.2%). Primary outcomes of therapy were as follows: complete response (CR), L group, *n* = 124 (40.7%) and H group, *n* = 89 (29.2%); partial response (PR), L group, *n* = 17 (5.6%) and H group, *n* = 26 (8.5%); progressive disease (PD), L group, *n* = 12 (3.9%) and H group, *n* = 15 (4.9%); and stable disease (SD), L group, *n* = 7 (2.3%) and H group, *n* = 15 (49%), respectively. The study population included 38.8% living and 61.2% dead patients, and 80.2% and 19.8% had residual disease or no residual disease (NRD), respectively. Ovarian tumors were unilateral, L group, *n* = 57 (16.1%) and H group, *n* = 44 (12.4%), and bilateral, L group, *n* = 122 (34.5%) and H group, *n* = 131 (37%), and 169 patients (44.9%) were aged >60 years and 207 patients (55.1%) ≤ 60 years.

### 3.2. Clinical Pathological Variables

**Figure 1:**
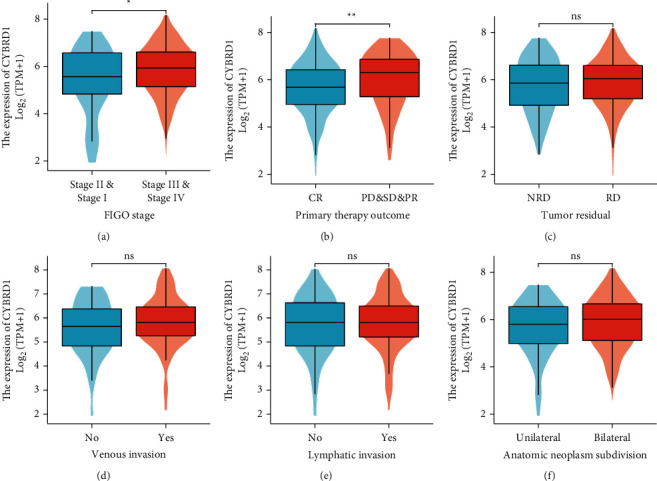
Correlation between CYBRD1 expression and clinicopathological characteristics of patients with ovarian serous cystadenocarcinoma (OV) obtained from TCGA. FIGO stage (a), CR/PD-SD-PR (b), residual disease (c), venous invasion (d), lymphatic invasion (e), and anatomical site (f). TCGA: The Cancer Genome Atlas.

High levels of CYBRD1 were significantly associated with the outcomes of primary therapy (SD-PD-PR versus CR, *P* < 0.05) and FIGO stage (I and II versus III and IV; *P* < 0.05) (Figures 1(a)–1(f)). Moreover, univariate logistic regression analysis revealed that high levels of CYBRD1 were significantly associated with poor outcomes of primary therapy (odds ratio [OR] = 0.719, CR versus PR-SD-PD), FIGO stage (OR = 1.471; I and II versus III and IV) (Table 2). These finding demonstrate that patients with OV with upregulation in CYBRD1 expression were more likely to develop a poorly differentiated tumor and a worse response to primary therapy.

### 3.3. Survival Outcomes and Multivariate Analysis

**Figure 2:**
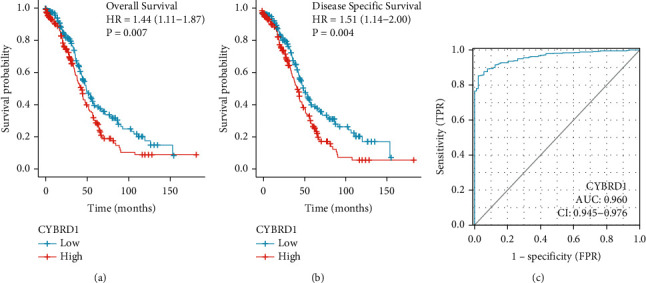
Kaplan–Meier analysis of the relationship between CYBRD1 expression and OS/DSS of TCGA patients with OV (a, b); the area under the ROC curve (AUC) of CYBRD1 (c). ROC: receiver operating characteristic curves; AUC: time‐dependent area under the curve.

Kaplan–Meier analysis and the log-rank test revealed that the high-CYBRD1 group experienced significantly shorter OS and DSS (Figures 2(a) and 2(b)). The area under the ROC curve (AUC) of CYBRD1 is 0.960 (95% confidence interval (95% CI): 0.945–0.976) (Figure 2(c)). This confirmed the good prognostic accuracy of CYBRD1. Univariate analysis revealed that high levels of CYBRD1 served as an independent factor that predicted shorter OS (HR, 1.438; CI, 1.107–1.868; *P*=0.007). The expression of CYBRD1 and primary therapy outcome and tumor residual were significantly associated with shorter survival (Table 3). Multivariate analysis revealed that CYBRD1 was significantly associated with OS (HR, 1.416; CI, 1.024–1.958; *P*=0.036) and the outcome of primary therapy (HR, 3.304; CI, 2.320–4.706; *P* < 0.001) (Table 3).

### 3.4. CYBRD1-Related Signaling Pathways and Functional Analysis

**Figure 3:**
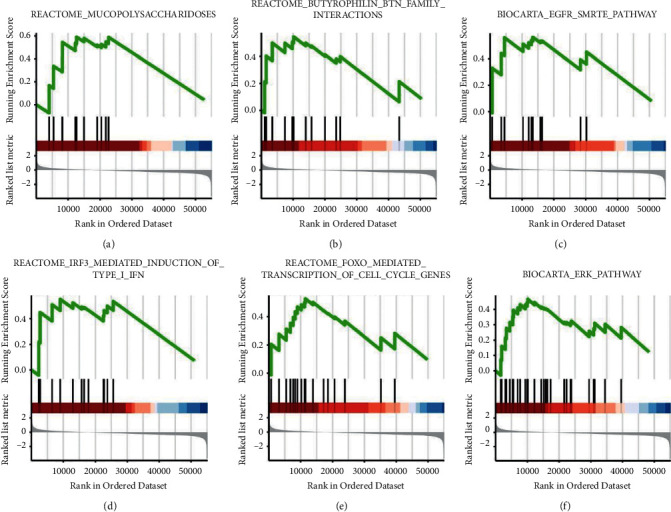
Gene set enrichment analysis (GSEA) of CYBRD1 in patients with OV from TCGA cohort. GSEA results demonstrating that mucopolysaccharidoses (a), butyrophilin BTN family interaction (b), EGFR/SMRTE pathway (c), IRF3-mediated induction of type I INF (d), FOXO-mediated transcription of cell cycle genes (e), and ERK pathway (f) were differentially enriched in CYBRD1-related EC. ES: enrichment score; NES: normalized ES; NOM *P* value: normalized *P* value.

To identify CYBRD1-related biological pathways involved in OV, we used GSEA (GSEA v2.0, http://www.broad.mit.edu/gsea/) to analyze pathways that significantly changed in OV samples (Figure 3 and Table 4). CYBRD1 levels (Figure 3 and Table 4) were significantly associated with mucopolysaccharidoses (NES = 1.749, NOM *P*=0.025; FDR, *P*=0.097) (Figure 3(a)), the butyrophilin BTN family (NES = 1.682; NOM *P*=0.023; FDR, *P*=0.095 (Figure 3(b)), the EGFR/SMRTE pathway (NES = 1.670, NOM *P*=0.025; FDR, *P*=0.097) (Figure 3(c)), IRF3-mediated induction of type I INF (NES = 1.698, NOM *P*=0.023; FDR, *P*=0.095) (Figure 3(d)), FOXO-mediated transcription of cell cycle genes (NES = 1.767, NOM *P*=0.011; FDR, *P*=0.078) (Figure 3(e)), and the ERK pathway (NES = 1.766, NOM *P*=0.008; FDR, *P*=0.07) (Figure 3(f)). These findings indicate that CYBRD1 was significantly associated with cell proliferation, energy metabolism, and apoptotic signaling pathways.

**Figure 4:**
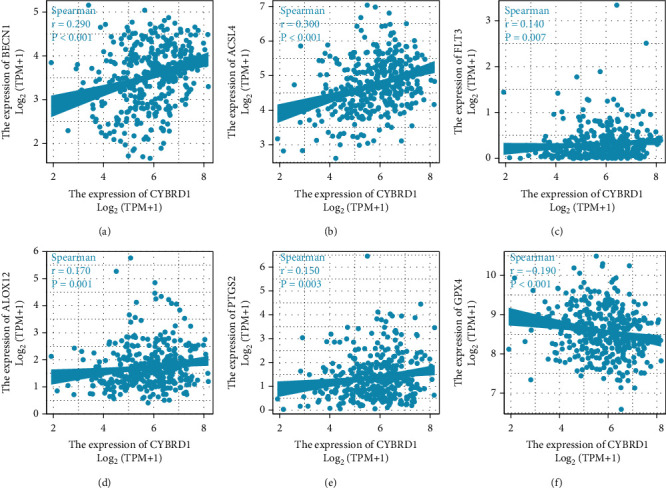
The correlation between CYBRD1 expression and ferroptotic biomarkers.

To identify CYBRD1 expression involved in ferroptosis, we analyzed the correlation between CYBRD1 expression and ferroptotic biomarkers. We found that BECN1 (Figure 4(a)), ACSL4 (Figure 4(b)), FLT3 (Figure 4(c)), ALOX12 (Figure 4(d)), and PTGS2 (Figure 4(e)) were positively correlated with the expression of CYBRD1 (*P* < 0.05). GPX4 (Figure 4(f)) were negatively correlated with the expression of CYBRD1 (*P* < 0.001). These findings indicate that CYBRD1 expression was significantly correlated with ferroptosis.

### 3.5. Immune Infiltration in OV

**Figure 5:**
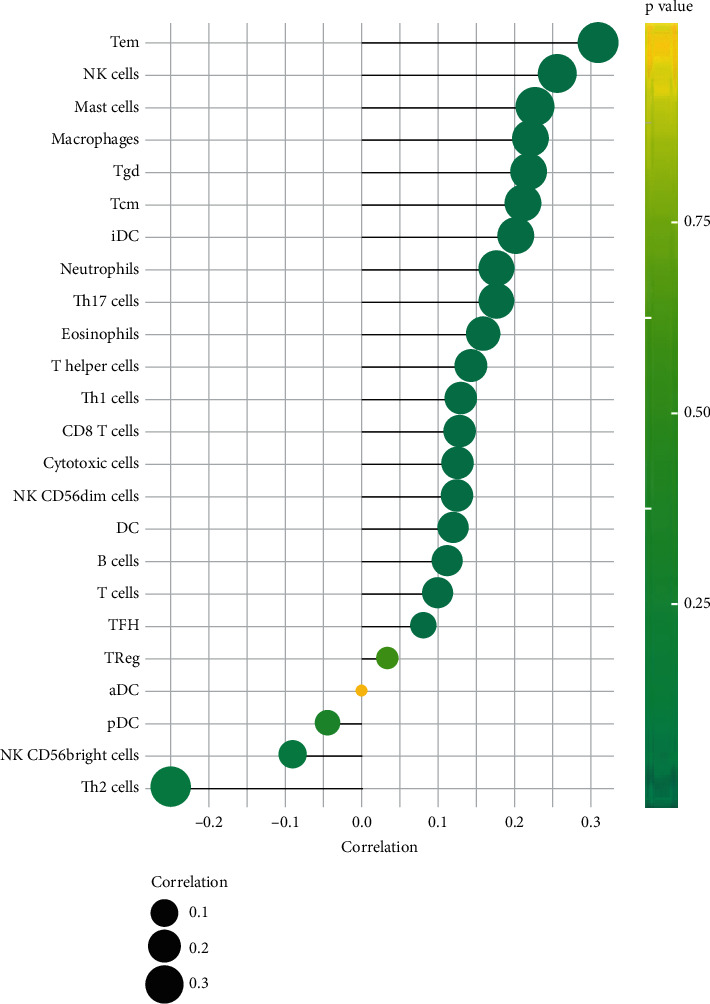
The correlation between the expression of CYBRD1 and immune-infiltration cells in OV from TCGA cohort.

The numbers of infiltrating T effector memory (Tem), natural killer (NKs), mast cells, macrophages, gamma delta T cells (*γδ* T cells), T central memory (Tcm), immature DCs (iDCs) neutrophils, T helper 17 (Th17) cells, eosinophils, T helper cells, T helper 1 (Th1) cells, CD8+ T cell, cytotoxic cells, NK CD56dim cells, dendritic cells (DCs), B cells, follicular helper (TFH), regulatory T (Treg) cells, and activated DCs (aDCs) were significantly and positively associated with high levels of CYBRD1 expression (Figure 5). The most highly positive correlations of CYBRD1 levels were with Tems, NK cells, and mast cells, and the most negative correlations were with CD56 bright NK cells and Th2 cells. The results showed that CYBRD1 was associated with immune infiltration in ovarian cancer. We speculated whether the upregulation of CYBRD1 expression could promote tumor progression through immune-related pathways. However, no other studies have shown that CYBRD1 can directly affect the prognosis of OV through immune mechanism. We only hypothesized and speculated through GSEA analysis, and the mechanism still needs to be further explored.

### 3.6. CYBRD1 Expression and Localization in Ovarian Tumor Tissues

**Figure 6:**
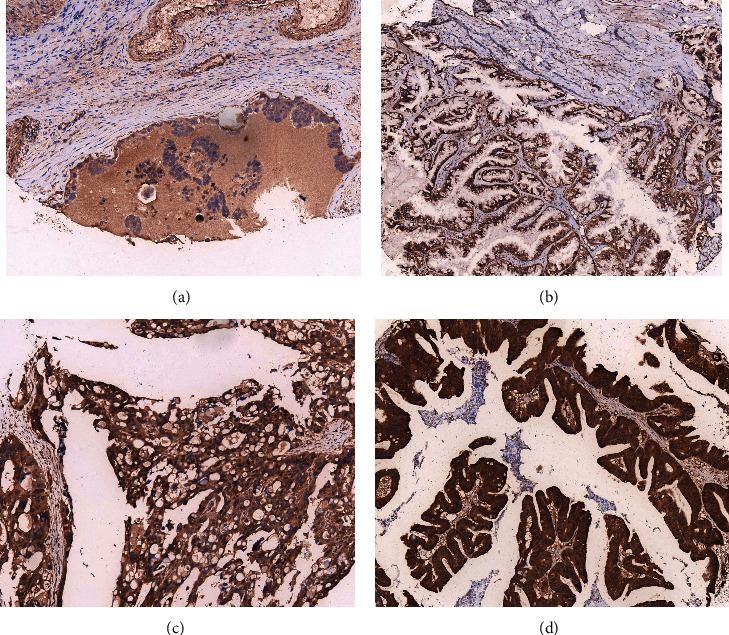
Immunohistochemical characterization of CYBRD1 expression of OV specimens from East Hospital (EH) cohort. Images of CYBRD1 protein compared the high and low expression of OV in EH cohort. Original magnifications ×200 (lower panels). 0, no staining (a); 1+, weak staining (b); 2+, moderate staining (c); 3+, strong staining (d). EH cohort: East Hospital cohort.

IHC was applied to measure the expression of CYBRD1 in OV tissues and verified CYBRD1 located within tumor cells and enriched predominantly in the cytoplasm of tumor cells (Figure 6). The 100 patients diagnosed with OV were divided into CYBRD1 low-expression group and high-expression group. We counted the clinicopathological characteristics in these patients (OS event, survival times, FIGO stage, lymphatic invasion, histologic grade, and age) (Table 5). Significant differences were identified for FIGO stage (OR = 3.69; I and II versus III and IV; *P*=0.004), histologic grade (OR = 7.14 G1&G2 versus G3&G4; *P* < 0.001), and lymphatic invasion (OR = 2.9,5 yes versus no; *P*=0.019) on the basis of the distinct CYBRD1 expression levels. However, CYBRD1 gene expressions were not statistically different in terms of age (OR = 1.45 age > 60 versus age ≤ 60; *P*=0.405) (Table 6).

### 3.7. Survival Outcomes and Multivariate Analysis

**Figure 7:**
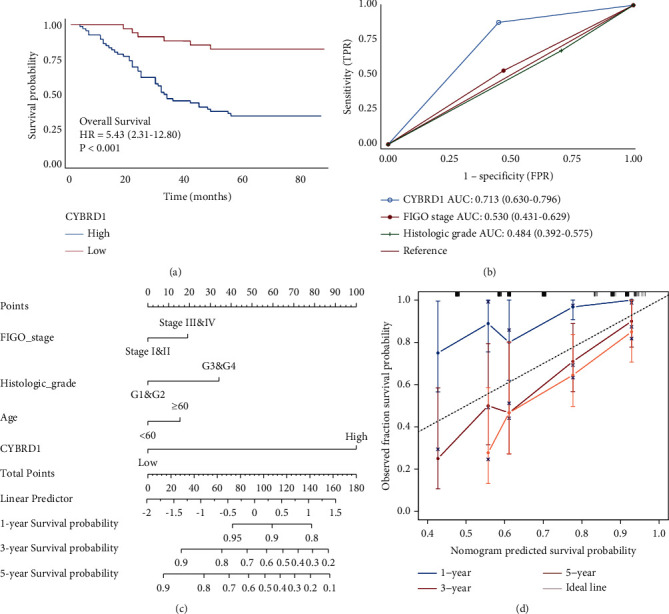
(a) Survival analysis of patients with OV from EH cohort according to CYBRD1 expression. Kaplan–Meier survival curves of OV patients for OS in CYBRD1^low^ and CYBRD1^high^ groups from EH cohort; (b) ROC curves with respect to the CYBRD1 expression, FIGO stage, and histological grade in patients with OV in EH cohort; (c) nomogram for CYBRD1 and other clinical characteristics in EH cohort OV patients, the C-index = 0.7016; (d) calibration analysis of the nomogram for 1-year OS, 3-year OS, and 5-year OS.

Kaplan–Meier analysis and the log-rank test revealed that patients in the CYBRD1high group experienced significantly shorter OS than patients in the CYBRD1 low group (HR = 5.43 (2.31–12.8), *P* < 0.001) (Figure 7(a)). We performed univariate and multivariate analysis to identify predictors of OS using the Cox regression model in the EH cohort (Table 7). Univariate analysis (HR, 5.43; CI: 2.31–12.80); *P* < 0.001) and multivariate analysis (HR, 8.42; CI: 3.24–21.89; *P* < 0.001) revealed that CYBRD1 was significantly associated with shorter OS (Table 7). We constructed a forest map of the risk score and clinicopathological parameters to identify the indicators that were significantly associated with OS. These parameters were included in the multivariate Cox regression model, revealing that CYBRD1 expression was independent risk factors associated with OS (Figure 7(b)). Subsequently, we estimated the efficiency of the predictive model to develop a quantitative approach for predicting the prognosis of OV patients. A nomogram that integrated the CYBRD1 and pathological variables was constructed, and the C-index = 0.7016 (Figure 7(c)). The bias-corrected line in the calibration plot was observed to be close to the ideal curve, which showed better consistency in terms of prediction and observation of the probability of the 3-year and 5-year OS than 1-year OS patients with OV (Figure 7(d)). This may be related to the small 1-year OS number of OV patients in EH cohort. All these findings suggest that the nomogram had a certain accuracy in predicting clinical outcome in OV patients.

## 4. Discussion

OV is the sixth most common genital malignancy of females worldwide and accounts for the highest mortality rate among gynecologic cancers. High-grade serous ovarian cancer is the most common histological subtype, accounting for 90% of cases [3]. Despite advances in basic research, chemotherapy, and surgery during the past 50 years, the morbidity and mortality rates of OV continue to increase [3].

Studies of the expression and functional activation of CYBRD1 were proposed [15, 24] because CYBRD1 mediates the transport of ferric ion in lung cancer cells and is involved in mitochondrial metabolism [24]. However, the relationship between CYBRD1 expression and immunocytes in OV is unknown. Here, we investigated the relationships between the expression of CYBRDR1, patients' clinical variables, and immune microenvironments of OV.

For this purpose, we conducted bioinformatics analyses of TCGA RNA-seq data. We found that high levels of CYBRD1 expression in OV were associated with worse outcomes of primary therapy, high histological grade, and poor prognosis. GSEA demonstrated that mucopolysaccharidoses, the butyrophilin BTN family, the EGFR/SMRTE pathway, IRF3- mediated induction of type I INF, FOXO-mediated transcription of cell cycle genes, and the ERK pathway were differentially enriched in association with high levels of CYBRD1 expression. These findings indicate that CYBRD1 may serve as a potential indicator of prognosis and a therapeutic target. Further, CYBRD1 expression was positively associated with the Tems, NK cells, and mast cells and was negatively associated with the numbers of CD56 bright NK and Th2 cells.

CYBRD1 is a ferrous ion-regulated reductase that activates multiple intracellular signaling pathways involved in transmembrane ferric ion transport [12]. CYBRD1 comprises 286 amino acid residues and six membrane-spanning domains [12]. The amino acid sequence of CYBRD1 is 45%–50% similar to that of cytochrome b561, which facilitates electron transport across the membrane [25]. CYBRD1 primarily acts as an iron- and hypoxia-regulated reductase, which is modulated by HIF-2*α* and inhibits the metabolism and absorption of iron [25]. Ferric iron is required for tumorigenesis and cancer progression [26]. Iron activates the generation of oxygen radicals, which contribute to cell death, ferroptosis, or carcinogenesis by directly damaging DNA [27].

CYBRD1 mediates direct electron transfer, instead of transport and diffusion across the membrane, and may therefore facilitate energy reprogramming in lung cancer epithelial cells [24]. Abnormal expression of CYBRD1 correlates with iron metabolism of TILs and may be regulated by activated HIF in malignant breast cells [28]. Moreover, CYBRD1 may serve as a prognostic marker for various cancers [28, 29]. For example, increased expression of plasma membrane-localized CYBRD1 is associated with favorable prognosis and is implicated in cancer cell proliferation and apoptosis in patients with breast cancer [15]. Further, a meta-analysis of TCGA data revealed that CYBRD1 expression is increased and serves as a prognostic indicator of patients with OV [29]. Similarly, our present study shows that high levels of CYBRD1 expression in OV were significantly associated with poor outcomes of primary therapy and FIGO stage.

Our present bioinformatics analyses revealed that CYBRD1 expression was associated with mucopolysaccharidoses, the butyrophilin BTN family, the EGFR/SMRTE pathway, IRF3-mediated induction of type I INF, FOXO-mediated transcription of cell cycle genes, and the MAPK/ERK signaling pathway, which are related to the proliferation and metastasis of OV cells. Further, activation of the FAK/ERK pathway contributes to tumor cell adhesion and the induction of ovarian cancer [30]. Others found that the IL-33/ST2 axis increases the growth of cancer cells via the MAPK/ERK/JNK signaling pathway and may serve as a prognostic indicator of patients with EOC [31].

Several studies illuminate the effects of signaling through the MAPK/ERK pathway associated with CYBRD1-mediated ion transport. For example, iron reduces the viability of OV cells when ERK signaling is altered [32]. Further, endometriosis-associated ovarian cancers exhibit a disequilibrium of iron homeostasis that is essential for the modulation of cell survival in a MAPK/ERK-dependent manner [33]. Moreover, secretory fimbrial epithelial cells exposed to iron enhance the proliferation of cancer cells, which is accompanied by changes in MAPK/ERK proteins [32].

A study of immune infiltration in patients with myelodysplastic syndrome with advanced clinical pathological features found that CYBRD1 expression regulates the cell cycle and DNA repair, whereas CD34 is downregulated and triggers an immune response [34]. Further, studies [4] found that OV is significantly affected by iron metabolism. Further research on the correlation between CYBRD1 expression, the ERK pathway, and immune infiltration is necessary.

Ferroptosis is a newly defined form of regulated cell death characterized by iron overload, lipid reactive oxygen species (ROS) accumulation, and activates MAPK signaling pathway to induce carcinogenesis, promote progression, and suppress immunity system [3, 35–37]. Hu et al. [38] found that the depletion of PIR initiates HMGB1-dependent autophagy by binding to BECN1 and subsequently promotes ferroptosis by activating ACSL4 in human pancreatic cancer cells. Yang et al. [39] identified that GPX4 modulate ferroptotic cancer cell death, and the upregulation of PTGS2 expression was a marker for lipid peroxidation in GPX4 induced ferroptosis in 17 types of cancers. Another report showed that the receptor tyrosine kinase Flt3 modulated glutamate oxidative stress-induced cell death, ROS production and lipid peroxidation in multiple neuronal cell lines, and primary cerebrocortical neurons [40]. Our findings showed that the CYBRD1 expression was significantly correlated positively with ACSL4, BECN1, PTGS2, ALOX12, and Flt3, which were “driver,” and negatively with GPX4, which was a “suppressor” in ferroptosis. Therefore, our findings suggested that ferroptosis may be one of the mechanisms of CYBRD1-mediated occurrence and development of OV.

Finally, we validated the correlation between CYBRD1 expression with prognostic factors in EH cohort. The results showed that CYBRD1 expression significantly enhanced in advanced stage (*P*=0.014), lymphatic invasion (*P*=0.017), and poor-differentiated tumor (*P* < 0.001). Moreover, CYBRD1 was an independent indicator of prognosis and ROC curves and the nomogram and calibration showed that CYBR1 had a certain accuracy in clinical prognostic prediction. Therefore, we can argue that CYBRD1 expression is significantly associated with short operating systems and acts as an independent predictor of adverse outcomes.

To our knowledge, the association of CYBRD1 in ovarian cancer has not been previously reported, and it will be helpful in clinical practice. There are some limitations in this study and lack of in-depth research on ferroptotic mechanisms, and the single validation may affect the accuracy and reliability of our results. Nevertheless, we believe that our findings are persuasive enough to ensure future studies with further clinical validation. At present, we only stay on the phenomenon research, we are not deep enough on the mechanism research, and we hope that the current problems found can play a certain helpful role in the future mechanism research.

## 5. Conclusions

Our findings indicate that CYBRD1 expression may serve as a novel prognostic indicator of poor outcomes of primary therapy and poor prognosis of patients with OV. Further, the ferroptosis and ERK pathway may be closely associated with CYBRD1 in OV. Moreover, our findings that CYBRD1 expression differentially correlated with the abundances of TILs and immune microenvironment. These results provide a platform for the development of novel inhibitors of the pathogenesis and progression of OV.

## Figures and Tables

**Table 1:** Characteristics of patients with ovarian serous cystadenocarcinoma obtained from TCGA data.

Characteristic	Low expression of CYBRD1	High expression of CYBRD1	*P*
*N*	188	188	

FIGO stage, *n* (%)			0.951
Stage I	1 (0.3%)	0 (0%)	
Stage II	11 (2.9%)	11 (2.9%)	
Stage III	145 (38.9%)	148 (39.7%)	
Stage IV	30 (8%)	27 (7.2%)	

Primary therapy outcome, *n* (%)			0.017
PD	12 (3.9%)	15 (4.9%)	
SD	7 (2.3%)	15 (4.9%)	
PR	17 (5.6%)	26 (8.5%)	
CR	124 (40.7%)	89 (29.2%)	

Age, *n* (%)			1.000
≤60	104 (27.7%)	103 (27.4%)	
>60	84 (22.3%)	85 (22.6%)	

Anatomic neoplasm subdivision, *n* (%)			0.201
Unilateral	57 (16.1%)	44 (12.4%)	
Bilateral	122 (34.5%)	131 (37%)	

Venous invasion, *n* (%)			0.623
No	25 (24.3%)	15 (14.6%)	
Yes	35 (34%)	28 (27.2%)	

Lymphatic invasion, *n* (%)			0.845
No	27 (18.2%)	21 (14.2%)	
Yes	53 (35.8%)	47 (31.8%)	

Tumor residual, *n* (%)			0.410
NRD	36 (10.8%)	30 (9%)	
RD	128 (38.4%)	139 (41.7%)	

OS event, *n* (%)			0.112
Alive	81 (21.5%)	65 (17.3%)	
Dead	107 (28.5%)	123 (32.7%)	

DSS event, *n* (%)			0.074
Alive	85 (24.2%)	67 (19.1%)	
Dead	91 (25.9%)	108 (30.8%)	

Age, median (IQR)	58 (50, 67)	59 (51, 68)	0.627

**Table 2:** The correlation of CYBRD1 expression and clinical pathological features in TCGA cohort (logistic regression).

Characteristics	Total (*N*)	Odds ratio (OR)	*P* value
FIGO stage (stage III and stage IV versus stage I and stage II)	376	1.471 (1.039–2.072)	0.027
Primary therapy outcome (CR versus PD, SD, and PR)	308	0.719 (0.567–0.899)	0.005
Venous invasion (yes versus no)	105	1.187 (0.837–1.702)	0.337
Anatomic neoplasm subdivision (bilateral versus unilateral)	357	1.181 (0.966–1.445)	0.104
Lymphatic invasion (yes versus no)	149	1.074 (0.802–1.435)	0.628
Tumor residual (RD versus NRD)	335	1.114 (0.876–1.435)	0.628
Age (>60 versus ≤60)	376	1.022 (0.680–1.535)	0.917

**Table 3:** Correlation between overall survival and clinicopathologic characteristics in TCGA patients applied Cox regression and multivariate survival model after variable selection.

Characteristics	Total (*N*)	Univariate analysis		Multivariate analysis	
		Hazard ratio (95% CI)	*P* value	Hazard ratio (95% CI)	*P* value
FIGO stage (stage III and stage IV versus stage I and stage II)	371	2.085 (0.925–4.699)	0.076	2.866 (0.696–11.791)	0.145
Primary therapy outcome (CR versus PD, SD, and PR)	304	4.280 (3.091–5.928)	<0.001	0.263 (0.185–0.374)	<0.001
Anatomic neoplasm subdivision (bilateral versus unilateral)	353	1.041 (0.768–1.410)	0.798		
Venous invasion (yes versus no)	103	0.905 (0.487–1.683)	0.753		
Lymphatic invasion (yes versus no)	147	1.422 (0.839–2.411)	0.191		
Tumor residual (RD versus NRD)	332	2.302 (1.479–3.583)	<0.001	1.589 (0.950–2.657)	0.077
Age (>60 versus ≤60)	374	1.373 (1.059–1.780)	0.017	1.314 (0.957–1.805)	0.092
CYBRD1 (high versus low)	374	1.438 (1.107–1.868)	0.007	1.319 (0.964–1.804)	0.083

**Table 4:** Gene sets enriched in the high-expressing CYBRD1 phenotype.

Description	Set size	Enrichment score	NES	*P* value	*P* adjust	q values	Rank	Leading_edge
BIOCARTA_ERK_PATHWAY	27	0.468	1.767	0.008	0.070	0.048	10123	Tags = 44%, list = 18%, signal = 36%
REACTOME_FOXO_MEDIATED_TRANSCRIPTION_O F_CELL_CYCLE_GENES	17	0.527	1.768	0.012	0.079	0.054	11438	Tags = 59%, list = 21%, signal = 47%
REACTOME_BUTYROPHILIN_BTN_FAMILY_INTERA CTIONS	12	0.546	1.682	0.024	0.096	0.066	10173	Tags = 50%, list = 18%, signal = 41%
REACTOME_IRF3_MEDIATED_INDUCTION_OF_TYP E_I_IFN	12	0.552	1.698	0.024	0.096	0.066	9022	Tags = 33%, list = 16%, signal = 28%
BIOCARTA_EGFR_SMRTE_PATHWAY	11	0.564	1.671	0.026	0.098	0.067	13402	Tags = 64%, list = 24%, signal = 48%
REACTOME_MUCOPOLYSACCHARIDOSES	11	0.591	1.749	0.026	0.098	0.067	12614	Tags = 55%, list = 23%, signal = 42%

**Table 5:** Characteristics of patients with OV obtained in EH cohort.

Characteristic	Low expression of CYBRD1	High expression of CYBRD1	*P*
*N*	34	66	

OS event			<0.001
Alive	28 (28%)	23 (23%)	
Dead	6 (6%)	43 (43%)	

Survival times, years			<0.001
<1	0 (0.0)	9 (9%)	
1–3	4 (4%)	27 (27%)	
3–5	9 (9%)	17 (17%)	
≥5	21 (21%)	13 (13%)	

FIGO stage, *n* (%)			0.014
Stage I	19 (19%)	23 (23%)	
Stage II	5 (5%)	3 (3%)	
Stage III	8 (8%)	35 (35%)	
Stage IV	2 (2%)	5 (5%)	

Lymphatic invasion, *n* (%)			0.017
No	25 (25%)	32 (32%)	
Yes	9 (9%)	34 (34%)	

Histologic grade, *n* (%)			<0.001
G1 + G2	20 (20%)	11 (11%)	
G3 + G4	14 (14%)	55 (55%)	

Age, *n* (%)			0.404
≤60	23 (23%)	39 (39%)	
>60	11 (11%)	27 (27%)	

**Table 6:** The correlation of CYBRD1 expression and clinical pathological features in EH cohort (logistic regression).

Characteristic	Total (*N*)	Odds ratio (OR)	*P*
FIGO stage (stages I and II versus FIGO stages III and IV)	100	3.69 (1.52, 8.97)	0.004
Histologic grade (G1 and G2 versus G3 and G4)	100	7.14 (2.79, 18.30)	<0.001
Age (age > 60 versus age ≤ 60)	100	1.45 (0.61, 3.46)	0.405
Lymphatic invasion (yes versus no)	100	2.95 (1.20, 7.27)	0.019

**Table 7:** Correlation between overall survival and clinicopathologic characteristics in EH cohort applied Cox regression and multivariate survival model after variable selection.

Characteristics	Total (*N*)	Univariate analysis		Multivariate analysis	
		Hazard ratio (95% CI)	*P* value	Hazard ratio (95% CI)	*P* value
FIGO stage (stage III and stage IV versus stage I and stage II)	100	1.14 (0.65, 2.00)	0.646	0.67 (0.37, 1.21)	0.185
Histologic grade (G1 and G2 versus G3 and G4)	100	1.09 (0.60, 1.98)	0.781	0.99 (0.54, 1.83)	0.984
Age (age > 60 versus age ≤ 60)	100	1.28 (0.73, 2.27)	0.391	0.49 (0.25, 0.93)	0.031
Lymphatic invasion (yes versus no)	100	1.33 (0.76, 2.33)	0.320	1.35 (0.73, 2.51)	0.342
CYBRD1 (high versus low)	100	5.43 (2.31, 12.80)	<0.001	8.42 (3.24, 21.89)	<0.001

## Data Availability

To analyze the roles of CYBRD1, RNA-seq data and relevant clinical data were downloaded from TGGA. There were all available data to be released.
